# Miniscule *Mollicutes*: Current hurdles to bacteriophage identification

**DOI:** 10.1093/sumbio/qvae019

**Published:** 2024-08-02

**Authors:** Jody Catchpowle, Julia Maynard, Barbara J Chang, Matthew S Payne, Michael L Beeton, Lucy L Furfaro

**Affiliations:** Medical School, The University of Western Australia, Perth, WA 6009, Australia; School of Biomedical Sciences, The University of Western Australia, Perth, WA 6009, Australia; School of Biomedical Sciences, The University of Western Australia, Perth, WA 6009, Australia; Medical School, The University of Western Australia, Perth, WA 6009, Australia; Microbiology and Infection Research Group, Cardiff School of Sport and Health Sciences, Cardiff Metropolitan University, Cardiff, CF5 2YB, Wales; Medical School, The University of Western Australia, Perth, WA 6009, Australia

**Keywords:** *Mollicutes*, bacteriophage (phage), Mycoplasma, Acholeplasma, Spiroplasma, Ureaplasma

## Abstract

*Mollicutes* are a diverse class of bacteria with a variety of unique characteristics that have allowed them to adapt to a range of hosts and often evade routine cultivation techniques. The focus of previous work has been on the major human pathogens; however, here we present a holistic introduction to the many other different genera that constitute the *Mollicutes*. They represent a significant One Health concern with limited available treatment options given their intrinsic and acquired resistance to many antibiotics. Bacteriophages (phages) are a promising therapeutic and one poorly explored in these bacteria and an avenue to understand gene transfer and resistance development. This review aims to emphasize the many unique and diverse qualities of the *Mollicutes* and synthesize our current understanding of phages of these bacteria and the challenges that have hindered their isolation and characterization.

**Sustainability Statement**

This review highlights the *Mollicutes* as an important class of organisms that contribute to many niches and have One Health implications. The UN sustainability goals addressed here, therefore include goals: 2, zero hunger; 3, good health and wellbeing; and 6, clean water and sanitation. The focus on the breadth of these microorganisms across the spectrum of human, animal, plant, and environmental health is complemented with the comprehensive review of the application of different techniques to assess bacteriophages and their role and future applications.

## The *Mollicutes*

**Figure 1.**
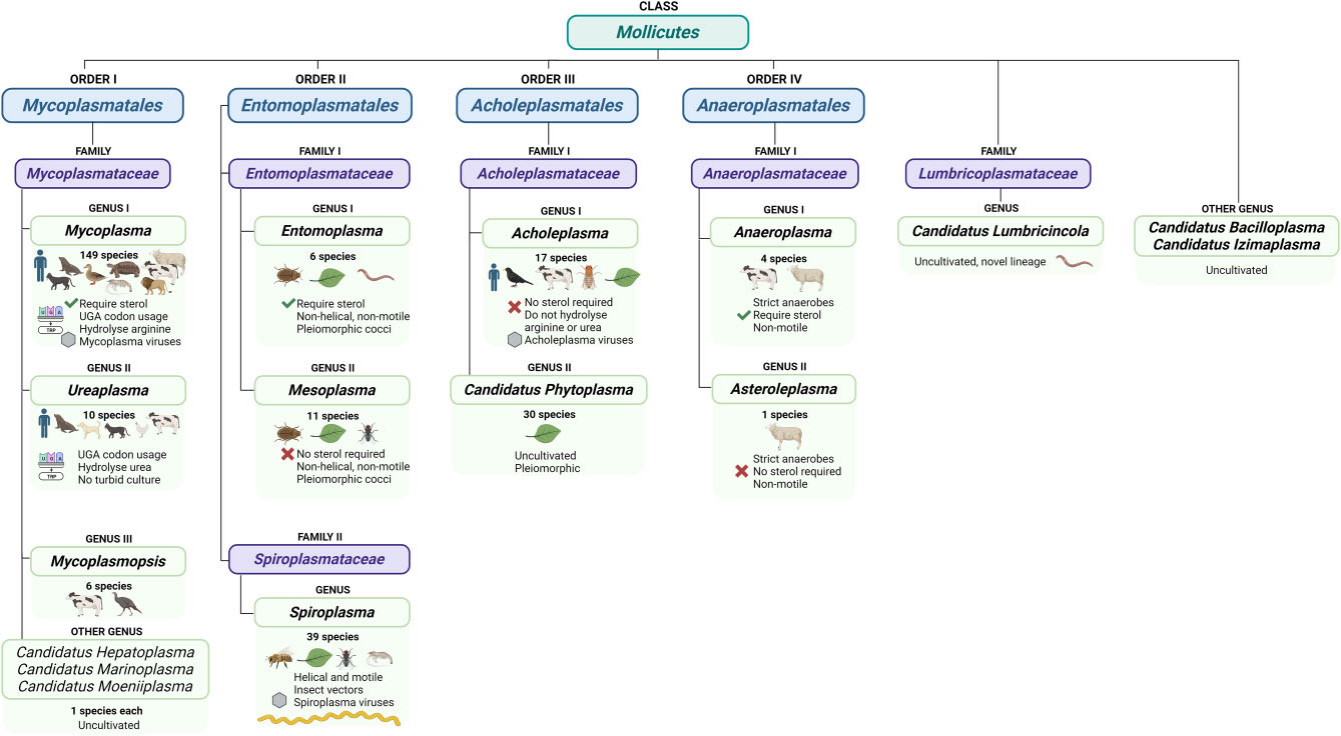
Diversity and unique features of the class *Mollicutes*.

*Mollicutes* are a class of bacteria consisting of 6 families, 15 genera, and 1494 species, according to the Encyclopaedia of Life (Parr et al. 2014) (Fig. 1). They are widely distributed in the environment and include many commensals but also several significant plant, animal, and human pathogens. *Mollicutes* are notoriously difficult to work with in a laboratory setting, and thus there remain gaps in our understanding of their biology. Considering their breadth of environmental niches, hosts, and disease presentation, they represent a One Health focus area that has been severely understudied to date.

The name *Mollicutes* originates from the Latin words *mollis*, meaning soft and *cutis*, meaning skin (Edward and Freundt 1967). *Mollicutes* are the smallest bacteria capable of self-replication and are characterized by their size (0.2–0.3 µm), small genome (577–2200 kb), lack of a typical bacterial cell wall, and reduced metabolic pathways (Pollack et al. 1997, Rivera-Tapia et al. 2002). For perspective, consider well-known *Escherichia coli* for comparison, a gram-negative rod 1–2 µm in length (0.5 µm width) and contains 2–10 times more genomic material (4500–5500 kb) (Riley 1999). These traits of *Mollicutes* lend themselves to unique characteristics, often including a parasitic lifestyle owing to their limited metabolic complexity, pleomorphic morphology derived from an atypical cell wall and low GC content ranging from 20 to 40% (Bové 1993). *Mollicutes* taxonomy has undergone review and suggested nomenclature changes have been proposed (Gupta et al. 2019) and debated (Balish et al. 2019); however, for clarity, this review will refer to the previous terminology as the most familiar to readers and the content covered (Parr et al. 2014). *Mollicutes* of orders *Mycoplasmatales* and *Entomoplasmatales* have evolved to use a unique genetic code (genetic code 4), with the universal UGA stop codon as a tryptophan codon instead (Brown et al. 2007, Ohama et al. 2008). They often have specific nutritional requirements; however, as auxotrophs, they lack the capability to produce their own, making them notoriously difficult to culture (Waites and Talkington 2004, Vega-Orellana et al. 2017). Most *Mollicutes* require sterols for *in vitro* culture, with *Ureaplasma* also requiring urea, and are often sensitive to changes in pH (Kenny and Cartwright 1977, Gaspari et al. 2020). *Mollicutes* are sensitive to detergents, osmotic shock, and are prone to dehydration, meaning extra care must be taken during sample collection. They are slow growing, with the doubling time of *Spiroplasma citri* being up to 36 h and *Mycoplasma* spp. from 0.5 to 8.3 h, compared to that of *E. coli*, which can be as low as 15 min (Beaman and Pollack 1983, Konai et al. 1996, Martínez-Torró et al. 2021). *Mollicutes* are a common laboratory contaminant in cell culture and are able to pass through filters commonly used to sterilize media (Tang et al. 2000). *Mollicutes* have evolved from gram-positive bacteria, are widely distributed in the environment, and show remarkable genetic diversity (Citti et al. 2018). This vast genetic diversity has made phylogenetic classification a challenge; with some species including the phytoplasmas remaining unculturable, and others such as *M. agalactiae* showing significant chromosomal rearrangement possibly from other species sharing the same host (Dworkin et al. 2006, Sirand-Pugnet et al. 2007a, Dordet-Frisoni et al. 2019). Their small genome and limited metabolic pathways led to hypotheses that these bacteria had undergone reductive evolution and were relatively simple. However, it has become increasingly evident that *Mollicutes* represent a complex class of microorganisms with significant gaps in our understanding that require further investigation. Advances have largely been hindered by the technical challenges of working with these unique organisms, and a new perspective to advance gold-standard microbiological assays will assist in determining their evolutionary path and role in health and disease.

## A one health issue

### Disease

Several *Mollicutes* are clinically and economically important for agriculture, veterinary, and human health. The significant human pathogens belong to the genera *Mycoplasma* and *Ureaplasma* and primarily exist on mucosal surfaces, including the urogenital and respiratory tract, where many are susceptible to complement-mediated lysis (Beeton et al. 2012). *Mycoplasma pneumoniae* is an important respiratory pathogen that causes upper and lower respiratory tract infections, atypical pneumonia, central nervous system disease, and *M. pneumoniae*-induced rash and mucositis (MIRM) (Waites and Talkington 2004, Meyer Sauteur et al. 2020). *Mycoplasma pneumoniae* has also been linked with asthma onset and exacerbation, as well as other chronic lung diseases such as chronic obstructive pulmonary disease (COPD) (Hong 2012, Amiri et al. 2018).

The remaining species relevant to human disease are primarily urogenital and perinatal opportunistic pathogens. *Mycoplasma hominis* is associated with bacterial vaginosis, pelvic inflammatory disease (PID), infertility, and pregnancy complications (Safa et al. 2018). Similarly, *M. genitalium* is associated with urethritis, PID, and adverse pregnancy outcomes (Lis et al. 2015). *Ureaplasma parvum* and *U. urealyticum* commonly colonize healthy individuals; however, are implicated in a variety of diseases, including urethritis, chorioamnionitis, preterm birth, and bronchopulmonary dysplasia in neonates (Paralanov et al. 2012, Beeton et al. 2019).

*Mollicutes* are also important in agriculture and include several plant and animal pathogens. *Spiroplasma citri* causes disease in citrus, carrots, and horseradish, and *S. kunkeli* causes corn stunt disease (Chen and Liao 1975). Both diseases are transmitted by insect vectors and have significant economic impacts, with anecdotal navel orange crop loss of 100% reported in one season in CA, USA, and 20–30% in Cyprus (EFSA Panel on Plant Health 2014, Rattner et al. 2021, Sagouti et al. 2022).

There are many *Mollicutes*, mostly *Mycoplasmas*, that are clinically relevant to domestic animals, including *M. gallisepticum* (birds), *M. hyorhinis*, and *M. hyopneumoniae* (pigs), *M. bovis* (cattle), *M. cynos* (dogs), *M. felis* (cats), and *M. arthritidis* (rodents). *Mycoplasma hyorhinis* and *M. hyopneumoniae* are significant pig pathogens with worldwide distribution, causing arthritis, respiratory disease, and poor weight gain in piglets (Martinson et al. 2018). Whilst economic evaluation is complex, in the USA, it is estimated that *M. hyopneumoniae* is responsible for annual losses of 400 million USD (Neumann et al. 2005). Similarly, *M. bovis* is a cause of bovine respiratory disease in cattle, along with mastitis and lameness, all of which are associated with significant economic burden (Pfützner and Sachse 1996). Treatment of *Mycoplasma* bovine respiratory disease in the USA was estimated at 50 million USD in 2011, excluding losses arising from mortality (Johnson and Pendell 2017). Experimental vaccines have been developed and to our knowledge three are currently commercially available for beef cattle; however, the efficacy for commercial vaccines varies (1–44%), and they are not currently useful for dairy cattle mastitis (Dudek et al. 2021, Prysliak et al. 2023). Metaphylactic antibiotics are still commonly used to prevent these diseases, which exacerbates the spread of antimicrobial resistance (Baptiste and Kyvsgaard 2017). The macrolides tulathromycin and gamithromycin are used to treat both bovine and porcine respiratory disease, and the fluoroquinolone enrofloxacin is used broadly for respiratory disease in domestic animals (Papich 2021). A major metabolite of enrofloxacin is ciprofloxacin, which is widely used in human medicine and its use is prohibited in some countries, such as Australia, in food production animals (Grabowski et al. 2022). Pigs treated with enrofloxacin have shown an increase in ciprofloxacin-resistant *E. coli* (Janssen et al. 2022). Furthermore, enrofloxacin has a long half-life and can be spread into the environment via the use of fertilizer, where it can persist for upwards of 3000 days (Trouchon and Lefebvre 2016). *Mollicutes* are evidently widespread in the environment, animals, and humans. This has relevance to the effective treatment options and resistance profiles of several pathogens.

### Current treatment and resistance

The primary treatments for *Mollicutes* are antibiotics; however, given their inherent resistance to several classes of antibiotics, this leaves a limited selection. *Mollicutes* are innately resistant to antibiotics that target cell wall synthesis, such as beta-lactams, bacitracins, and glycopeptides (Chernova et al. 2021). They are also resistant to drugs that target folic acid synthesis and to polymyxin A and rifampin. The most effective drugs are macrolides, tetracyclines (inhibit protein synthesis), and quinolones (inhibit DNA replication). *Mollicutes* are susceptible to several other antibiotics, including daptomycin, chloramphenicol, and aminoglycosides; however, susceptibility does vary from species to species (Chernova et al. 2021).

Macrolides are a primary treatment for *Mollicutes* and resistance is increasingly observed to this class of drug (Lysnyansky and Ayling 2016). The frequency of resistance to macrolides varies geographically, and in parts of Asia, >90% of *M. pneumoniae* strains exhibit resistance (Pereyre et al. 2016). This resistance is mostly related to chromosomal mutations, particularly in domain V of the peptidyl-transferase loop and the domain II hairpin 35 of the 23S rRNA (Vester and Douthwaite 2001, Pereyre and Tardy 2021). Only a few mutations are required for resistance to occur via the 23S rRNA peptidyl-transferase loop due to *Mycoplasmas* only having one or two rRNA operons (Pereyre and Tardy 2021). Resistance is also found in the other commonly used classes of antibiotics, quinolones, and tetracyclines (Pereyre and Tardy 2021). Mechanisms of resistance include mutations in DNA gyrase and topoisomerase IV target genes, and a combination of mutations in both genes results in a high level of resistance. The presence of *tet(M)* genes is responsible for high levels of resistance to tetracyclines in *M. hominis* and *Ureaplasma* spp. With the Tet(M) protein causing conformational ribosome changes that interfere with tetracycline binding (Chernov et al. 2018, Pereyre and Tardy 2021). In human treatment, it is notable that often both tetracycline and fluoroquinolones are contraindicated during pregnancy and for young children, resulting in even fewer options for this population (Kline et al. 2012, Bookstaver et al. 2015). There is one fluoroquinolone, tosufloxacin, i.e. approved for use in children in Japan and may provide a suitable treatment option in cases of macrolide resistance; however, it is associated with risk of kidney damage (Kawai et al. 2013, Atsumi et al. 2022).

Whilst most resistance is conferred by point mutations, horizontal gene transfer (HGT) is more important for *Mollicutes* than previously thought. HGT involves the transfer of large portions of genetic material via mobile genetic elements (MGEs), such as plasmids and prophages. HGT was thought to be minimal in *Mollicutes* because of their small genome; however, evidence suggests that they are more sophisticated than previously thought and are capable of extensive HGT (Faucher et al. 2019).

## Genetic transfer and exchange

Although *Mollicutes* genomes share several features, they lack a common genome organization (synteny), which is part of the mosaic nature of these genomes (Sirand-Pugnet et al. 2007a). An important mechanism for bacterial evolution, HGT is a significant contributor to the genetic diversity of *Mollicutes*. Citti et al. reviewed the extensive use of HGT, with a focus on conjugation. Conjugation requires the donor and recipient cells to be in contact and involves the transfer of genetic materials by integrative and conjugative elements (ICEs) or plasmids (Citti et al. 2018). Mycoplasma ICEs (MICEs) are able to randomly integrate into the host chromosome, contributing to mosaicism, and encode a type IV secretion system that mediates transfer via conjugation. MICEs have been found in several species, including *M. agalactiae* and *M. fermentans* (Citti et al. 2018). However, MICEs appear to be absent from several important species, such as *M. pneumoniae* and *M. genitalium* (Tardy et al. 2015). Another type of conjugative process observed in several species is mycoplasma chromosomal transfer (MCT), which requires the presence of an ICE in the recipient but not the donor cell, and results in the transfer of large genomic segments by homologous recombination (Ana et al. 2022, Dordet-Frisoni et al. 2022). This unconventional conjugative mechanism is thought to have a great impact on the evolution and adaptability of the mycoplasmas, contributing to the high genome plasticity and mosaicism of these bacteria (Dordet-Frisoni et al. 2019, Garcia-Galan et al. 2022).

It is possible that extracellular vesicles (EVs) could act as vehicles to transfer such genes; the role of EVs in HGT has been reported in a number of prokaryotes (Domingues and Nielsen 2017). Several *Mycoplasma* species have been found to produce EVs when nutritionally stressed (Gaurivaud et al. 2018). Budding/vesicles have been observed in *U. parvum* whilst using scanning electron microscope (SEM) to observe biofilms (Gaurivaud et al. 2018, Rowlands et al. 2021). In one report, *Acholeplasma* EVs were shown to mediate transfer of mutant genes involved in quinolone resistance (Medvedeva et al. 2014). The role of EVs in genetic exchange and the spread of antibiotic resistance among *Mycoplasma* species deserve further study.

Through HGT, *Mollicutes* have become highly adaptable, and this is evident from the number of host species they inhabit. Niche adaptation of these bacteria is suggested to occur *in situ* through the cross-species sharing of genetic material to improve fitness in the new environment. This alone has serious implications for the continuing evolution and host expansion of these organisms in the context of One Health emerging pathogens. While mobile genetic elements such as ICEs and genomic islands have been detailed (Citti et al. 2020), phages and transduction have been less so.

### Bacteriophages

There is a limited arsenal of antibiotics available to treat *Mollicute* disease due to intrinsic resistance, with acquired resistance becoming an imminent concern. Few new antibiotics are being developed and alternatives to antibiotics are needed to reduce their use and tackle increasing antimicrobial resistance.

A promising alternative treatment of interest is the use of bacteriophages. Bacteriophages (phages) are abundant viruses that can infect specific bacteria. They then proceed with one of several lifecycles. The phage can replicate inside the bacterium and then lyse the cell; this is known as the lytic cycle. Obligately lytic phages provide certain therapeutic advantages over traditional antibiotics as phages are highly specific to their host, are self-dosing, have good safety profiles, and, in many cases, are readily isolated from the environment. Phages are not a new discovery and were first observed in 1915 (Twort 1915, d'Herelle 1917). However, the discovery of antibiotics and their use in World War II meant that the Western world largely abandoned therapeutic phage research. Former Soviet countries, however, continued using phage therapy, and it is still commonly used today (Chanishvili et al. 2012). In the wake of increasing antimicrobial resistance, there is renewed interest in phage therapy in the Western world (Kakasis and Panitsa 2019).

There are several obstacles to using phages as a therapy. Regulation is challenging as personalized phage cocktails are required and this does not easily fit the current therapeutic drug approval model (Furfaro et al. 2018, Petrovic Fabijan et al. 2023). Further, finding the right phages can be challenging and phages must be screened to ensure they do not carry unwanted antimicrobial resistance (AMR) or virulence genes (Pires et al. 2020). Therapeutic use of phages also currently relies on the killing activity that obligately lytic phages possess and therefore phage therapy candidates must be screened for this property (Venturini et al. 2022).

As mentioned, there are other lifecycles phages may undertake, which are not always as straightforward as the lytic cycle would indicate. Some phages may enter a lysogenic lifecycle whereby they integrate into the host genome and do not kill the infected cell, forming a lysogen. These temperate phages will then replicate as the bacteria replicates, with external stressors or spontaneous events triggering excision from the genome (termed induction) into a lytic cycle. Alternatively, other phages may enter a chronic infection lifecycle whereby virions are released from the host cell, but lysis does not occur; this includes budding or extrusion. Chronic infection can occur with or without lysogeny (Hobbs and Abedon 2016). Phages capable of infecting *Mollicutes* can utilize chronic lifecycles, which allow the virus to be lysogenic whilst also causing a productive infection. These lifestyles are not extensively researched in *Mollicutes* for several reasons relating to difficulties culturing and observing phage activity; here, we cover the rarely described exit strategies of *Mollicutes* phages.

### Budding

The budding process (gemmation) is a unique feature of pleomorphic phage, with an external lipid bilayer, represented by *Plasmaviridae* (Drulis-Kawa et al. 2015). Budding can occur in *Mollicutes* because they have an atypical cell wall. Amongst phages characterized to date, lipid-containing phages are considered relatively rare. This may be partly due to biases inherent in common techniques used to isolate phages, such as the use of chloroform. However, for lipid-containing phages that have been characterized, it appears that the lipids play a significant role in their lifecycle. In *Plasmaviridae*, lipids are obtained from their host in an unselective process during virion release, as the phages do not have genes to produce their own (Atanasova et al. 2015).

Two lipid-containing phages capable of infecting *Mollicutes* that have been identified. L2 and L172 phages are pleomorphic, lipid-containing enveloped viruses that can infect *A. laidlawii* (Liss and Ritter 1985). These phages have been shown to cause persistent, non-lytic, productive infections. It is thought that L2 and L172 non-selectively acquire their membrane from the bacterial host during budding (Al-Shammari and Smith 1981). It was initially reported that the two viruses were not related, although the method used (hybridization) would not have allowed a detailed comparison (Dybvig et al. 1985). However, L2 has a double-stranded DNA genome, while L172 is a single-stranded DNA virus. As the sequence of L172 is not available, further assessment of their evolutionary relationship is not yet possible, and L2 remains the only species of *Plasmaviridae* (Krupovic and Consortium 2018).

The viruses produced by L2 are heterogenous, with the distinct morphological subtypes of viruses being produced with different sedimentation velocities (Poddar et al. 2008). They were found to contain the same proteins, but in varying amounts. Similarly, while containing the same genome, some virions contained up to three copies.

A budding-like mechanism has also been observed for phages L3 (*A. laidlawii*), SpV3 (*S. mirum*), and ai (*S. citri*); however, the release of numerous virions can lead to degradation of the bacterial cell and result in eventual death of the host bacterium (Drulis-Kawa et al. 2015). Unlike traditional enzymatic host cell lysis, this process occurs more slowly, thus maintaining the host membrane integrity for longer. From a therapeutic viewpoint, budding phages would not represent an ideal phage therapy candidate; however, there is a deficit in understanding of alternative phage infection strategies (Mäntynen et al. 2021). As phages with lipid membranes enter the cell via fusion of the membranes, it may be hypothesized that the virions may have a broad host range, able to infect the majority of strains within that species and perhaps beyond. This lifestyle may also be an attractive option for drug delivery options in the future through this encapsulated approach.

### Extrusion

**Figure 2.**
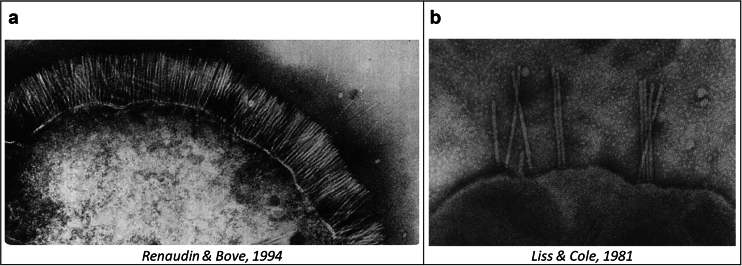
Examples of *Mollicutes* phages visualized by electron microscopy. (a) *Spiroplasma* phage SpV1 extruding from the membrane of *S. citri* host at 60000× magnification (Renaudin and Bove 1994) and (b) *Spiroplasma* virus (Group 1 phages) on the *S. melliferum* BC3 host at 250000× magnification (Liss and Cole 1981).

Extrusion is another chronic phage exit strategy that *Inoviridae* undertake, whereby phages are assembled and secreted via channels at the cell membrane (Hay and Lithgow 2019). Filamentous phages, which establish a chronic infection, can be found to decorate the host bacteria, appearing as rods protruding from the surface of the host cell (Fig. 2). Plectroviruses, such as *Spiroplasma* phage SpV1, adsorb to specific cell surface receptors, which are often plasmid-encoded conjugative pili, then bind to the periplasmic protein co-receptors that couple adsorption with DNA uptake into the cytoplasm (Liss and Cole 1982). Following infection, it has been observed that while viral titres increased, indicating productive infection and replication, the rate of increase in bacterial counts remained the same across infected and non-infected cells. This supports the filamentous nature of these phages and suggests non-lytic productive infection. This chronic infection is another example of effective transmission of phage genomic material without resulting in bacterial death. Other examples of filamentous phages include MV-L51 (L51), which infects host *A. laidlawii* and was isolated from an extremely rare spontaneous plaque (Liss and Maniloff 1973). Given the lack of cell lysis caused by filamentous phages, they are not frequently detected by plaque assays, unlike many obligately lytic phages and temperate phages (Knezevic et al. 2021).

### Bacteriophages of *Mollicutes*

Researchers have isolated phages from *Mollicutes* from as far back as the 1970s (Table 1). However, due to the difficulties associated with culturing some *Mollicutes*, such as *Ureaplasma*, phage isolation has remained elusive in certain species. Advances in molecular methods have allowed phage-related sequences to be identified within the genomes of some species; however, isolation of the virions largely remains a challenge.

**Table 1.** Bacteriophages of *Mollicutes* and their features.

	Phage name(s)	Bacterial host	Source/Niche	Genome	Accession	Taxonomy	Comments	Reference
**Acholeplasma phages**	**L1** (MV-L1, L51, MV-51, AV-L51)	*A. laidlawii*	Bovine	ssDNA 4.491 kb circular	X58839	*Plectroviridae, Plectrovirus*	Filamentous, helical, no envelope, bullet-shaped, 12–16 × 70–90 nm, release via extrusion. First isolation of a *Mycoplasma* virus (1970). Turbid plaques. L51 subsequently isolated from a spontaneous plaque, equivalent to L1.	Gourlay (1970) and Liss and Maniloff (1973)
	**L2** (MV-L2, AV-L2)	*A. laidlawii*	Various animals	dsDNA 11.6 kb circular superhelical	NC_001447	*Plasmaviridae, Plasmavirus*	Enveloped, quasispherical, 50-125 nm, *Plasmaviridae* (budding). L2 is the type member of *Plasmaviruses*. Turbid plaques.	Gourlay (1971)
	**L3** (MV-L3, AV-L3)	*A. laidlawii, A. modicum*	Various animals	dsDNA 39 kb linear, circularly permuted, terminally redundant	–	*Caudoviricetes* (formerly *Podoviridae*)	Short-tailed phage, isometric head, no envelope, ∼60 nm diameter, and a short tail ∼10 × 20 nm. Clear plaques.	Garwes et al. (1975) and Haberer and Maniloff (1982)
	**L172** (MV-L172, AV-L172)	*A. laidlawii*	Various animals	ssDNA 14 kb circular	–	Unclassified	Enveloped, quasispherical, globular, pleomorphic, 60−80 nm in diameter virion with no detectable DNA homology to L2 phage.	(Liska 1972)
	**M1**	*A. modicum*	Various animals	–	–	*Plasmaviridae, Plasmavirus*	Enveloped, quasispherical, 105−160 nm, sensitive to chloroform. Isolated from spontaneous plaques.	Congdon et al. (1979)
	**O1** (MV-O1)	*A. oculi*	Goat	DNA	–	*Plasmaviridae, Plasmavirus*	Enveloped, quasispherical, 80–130 nm. Isolated from spontaneous plaques.	Ichimaru and Nakamura (1983) and Ogawa and Nakamura (1985)
**Mycoplasma phages**	**Br1**	*M. bovirhinis*	Cow	–	–	*Caudoviricetes* (formerly *Myoviridae*)	Long-tailed phage, isometric head, long contractile tail, no envelope, Br1 is a typical contractile-tailed bacteriophage with a head 77 nm in diameter and a tail 104 nm long.	Gourlay et al. (1983a)
	**Hr1**	*M. hyorhinis*	Pig	DNA	–	–	Short-tailed phage, isometric head, no envelope, *Mycoplasma* virus Hr1 is a short-tailed bacteriophage with a polyhedral head about 34 nm across and a tail about 14 nm long. It produces plaques on some strains of *M. hyorhinis*.	Gourlay et al. (1983b)
	**P1**	*M. pulmonis*	Rodent	dsDNA 11.66 kb linear	NC_002515	*Caudoviricites, Delislevirus*	Short-tailed phage, isometric head, no envelope.	Zou et al. (1995)
	**MHoV-1**	*M. hominis*	Humans	dsDNA 15.9 kb	CP009652	–	Proviral, prophage genome related to *M. fermentans* MFV-1 and *M. arthritidis* MAV-1.	Calcutt and Foecking (2015)
	**MAV1** (MAV-1)	*M. arthritidis*	Rodent	dsDNA 15.644 kb linear	NC_001942	–	Temperate, MAV1 harbours an important pathogenesis factor of *M. arthritidis*-induced arthritis of rats. Two known hosts.	Voelker et al. (1995)
	**MGyu-2021a**	*M. anserisalpingitidis*	Goose	DNA 24.404 kb linear	MW358880	Unclassified	Isolated from goose cloacae, *M. anserisalpingitidis* causes inflammation of the genital tract and cloaca and leads to losses from reduced egg production. One known host.	Grózner et al. (2021)
	φ**MFV1** (MFV-1)	*M. fermentans*	Human	DNA 18.855 kb linear	NC_005964	Unclassified	Prophage, one known host.	Röske et al. (2004)
**Spiroplasma phages**	**SPIROPLASMA GROUP 1** (SpV1, SVC1, C1)							
	**SV1** (SpV1, C1)	*S. melliferum, S. citri*	Honeybee (insect)/Plants	ssDNA	–	*Plectroviridae, Plectrovirus*	Filamentous, no envelope, 10–15 × 230–280 nm, C1-like viruses, originally identified by electron microscopy. Turbid plaques.	Cole et al. (1973a,b)) and Liss and Cole (1981)
	**C74 **(SpV1-C74)	*S. citri, S. melliferum*	Plant/Insect	ssDNA 7.768 kb circular	U28974	*Plectroviridae, Vespertiliovirus*	Filamentous, rod-shaped, no envelope.	Bébéar et al. (1996)
	**R8A2B** (SpV1-R8A2B)	*S. citri*	Plant	ssDNA 8.273 kb circular	X51344	*Plectroviridae, Vespertiliovirus*	Type 1 *Spiroplasma* virus, naked, rod-shaped virus.	Renaudin et al. (1990)
	**SkV1CR23x**	*S. kunkelii*	Plant	ssDNA 7.870 kb circular	EF506570	*Plectroviridae, Vespertiliovirus*	Rod-shaped, no envelope, extrusion.	Zhao et al. (2003)
	**aa** (1-aa)	*S. citri*	Plants	ssDNA 8.5 kb circular	–	*Plectroviridae*, *Plectrovirus*	Filamentous, rod-shaped, no envelope, 10–15 × 230–280 nm, type 1-like *Spiroplasma* viruses. Small turbid plaques.	Dickinson and Townsend (1984a)
	**SVGII-3** (SVGII3)	*S. citri*	*Circulifer haematoceps* (plant insect)	ssDNA 7.878 kb circular	NC_073761	*Plectroviridae, Virgulavirus*	Type 1 *Spiroplasma* virus.	Foissac et al. Replicative form of a SVGII-3 type plectrovirus detected in *Spiroplasma citri* strain GII3. Unpublished, direct submission.
	**SVTS2**	*S. melliferum*	Honeybee (insect)	ssDNA 6.825 kb circular	AF133242	*Plectroviridae, Suturavirus*	Type 1 *Spiroplasma* virus, only species in the *Suturavirus* genus.	Sha et al. (2000)
	**SPIROPLASMA GROUP 2** (SpV2, SVC2, C2)							
	**C2** (SpV2, SVC2)	–	–	–	–	*Plectroviridae, Vespertiliovirus*	Isometric head and long non-contractile tail, cryptic ‘phage-like’ particles.	Cole et al. (1973a,b)
	**SPIROPLASMA GROUP 3** (SpV3, SVC3, C3)							
	**SVC3** (C3)	*S. citri*	Plants	dsDNA 21 kb linear	–	*Caudoviricetes* (formerly *Podoviridae*)	Short-tailed phage, isometric head, no envelope. Clear plaques.	Cole et al. (1977), Cole et al. (1974), Cole (1977))
	**SVC3/608**	*S. citri*	Plants	dsDNA 21 kb linear	–	–	Membrane-bound budding virions, short-tailed phage, isometric head, no envelope, type 3-like viruses.	Cole et al. (1973a,b)
	**SVC3/SMCA**	*S. mirum* and *S. citri*	Animal/Plants	dsDNA 21 kb linear	–	–	Membrane-bound budding virions, short-tailed phage, isometric head, no envelope, type 3-like viruses.	Cole et al. (1977)
	**ag** (SpV3-ag)	*S. citri*	Plants	dsDNA 21 kb linear	–	–	Short-tailed phage, isometric head, no envelope, type 3-like viruses.	Dickinson et al. (1984)
	**AV9/3**	*S. citri*	Plants	dsDNA 21 kb linear	–	–	Short-tailed phage, isometric head, no envelope, type 3-like viruses.	Dickinson et al. (1984)
	**ai** (SpV3-ai)	*S. citri*	Plants	DNA 16 kb linear (cohesive ends)	–	–	Short-tailed, polyhedral, type 3 *Spiroplasma* virus, temperate.	Dickinson and Townsend (1984b)
	**SRO viruses**	*Spiroplasma* SROs	*Drosophilia* species (fly)	17, 21.8, >30 kb, Linear	–	Unclassified	Isometric head, short-tailed.	Oishi and Poulso (1970) and Oishi (1971)
	**SpV3**	*S. mirum*	*Drosophila willistoni* (Insect)	21.8 kb	–	–	Short-tailed polyhedron, budding release. Sex-ratio organism (SRO) virus, causing elimination of males from the reproductive infected females.	Cohen et al. (1987)
	**NSRO-P1**, **NSRO-P2**, **MSRO-P1**	*S. poulsonii*	*Drosophila* species (fly)	DNA 18.9–19.7 kb linear	OL689227, OL689226, OL689230	Unclassified	SRO viruses. Endosymbiont of Drosophila.	Ramirez et al. (2022)
	**SPIROPLASMA GROUP 4** (SpV4, SVC4, and C4)							
	**SpV4** (SVC4, C4)	*S. melliferum*	Honeybee (Insect)	ssDNA 4.421 kb Circular	NC 003438	*Microviridae, Spiromicrovirus*	*Microviridae* SpV4, is a small, non-enveloped lytic virus that infects the helical *Mollicutes* and *S. melliferum*.	Ricard et al. (1980)

Phages that can infect *Ureaplasma* have not yet been identified; however, phage genes have been reported. Phage genes were identified in 9 out of 14 *U. parvum* and *U. urealyticum* serovars sequenced by Paralanov and colleagues (2012). The identified genes comprised those encoding an integrase-recombinase protein, phage recombination protein, and phage terminase. This suggests a temperate phage insertion, with the authors positing it may be a pathogenicity island due to its presence/absence within samples of the same serovar. Further, the presence of restriction modification (RM) systems suggests a need for *Ureaplasma* to defend itself from foreign viruses.

Prophages have been identified in the genomes of *M. hominis* and *M. fermentans*. The *M. hominis* prophage, named MHoV-1, is a 15.9 kb sequence i.e. related to *M. fermentans* MFV-1 prophage and the rodent pathogen *M. arthritidis* MAV-1 phage (Calcutt and Foecking 2015). It is noteworthy that only a handful (25) of *M. hominis* genomes had been sequenced and uploaded until 2020; however, in the last few years, this number has increased to 63 in the NCBI database, providing an opportunity to search for phages (Jin et al. 2020). MFV-1 is a prophage with a sequence of ∼16 kb. It is believed to be mobile and encodes a surface membrane protein (Röske et al. 2004). It is suggested that these prophages are related and were acquired before branching of *Mycoplasma* clades. Neither has yet been reported to produce infective plaques. However, MAV-1 has been shown to be infective and is related to both MHoV-1 and MFV-1. MAV-1 and MFV-1 are predicted to have similar functions.

**Figure 3.**
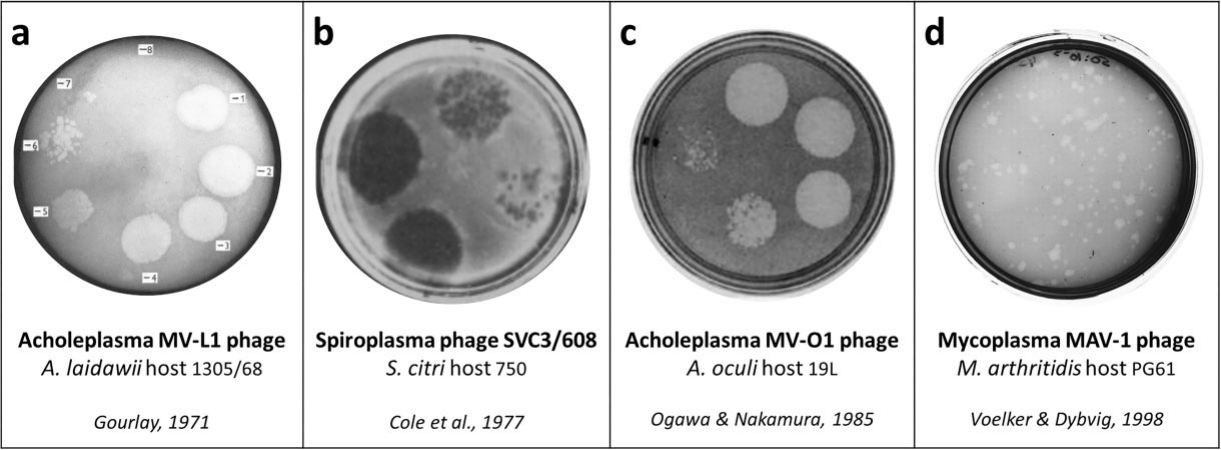
Examples of *Mollicute* bacteriophage activity including examples from the literature of clearing zones and/or plaques on lawns. (a) *A. laidlawii* (Gourlay 1971), (b) *S. citri* (Cole et al. 1977), (c) *A. oculi* (Ogawa and Nakamura 1985), and (d) *M. arthritidis* (Voelker and Dybvig 1998).

MAV-1 is a temperate phage of *M. arthritidis* that was isolated in 1994 (Voelker et al. 1995). It is a 16.5-kb double-stranded linear DNA phage, which may be required for *M. arthritidis* to cause disease in rodents. The original methodology is, to our knowledge, unpublished; however, subsequent papers imply that MAV-1 was spontaneously induced from *M. arthritidis* strain 158p10. The phenotype of MAV-1 was able to be characterized with plaques observed on 4/12 strains of *M. arthritidis* tested (Voelker and Dybvig 1998). Plaques appeared clear, but on closer inspection with an inverted microscope colonies could be seen within the zone of clearing (Fig. 3d). MAV-1 was unable to be propagated in a broth and attempts at induction with mitomycin C were unsuccessful. It was also found to be Proteinase K resistant, which is uncommon; however, interestingly SpV4 phage, which can infect *S. citri* is also resistant to Proteinase K. MAV-1 is resistant to chloroform, indicating it does not contain lipids and therefore does not produce virions via budding.

Hr1 is a further example of a temperate phage, spontaneously induced from a strain of *M. hyorhinis* in 1983 (Gourlay et al. 1983). Hr1 was able to produce clear plaques of up to 2 mm in diameter on a lawn of *M. hyorhinis* but not on *A. laidlawii* or *M. bovirhinis*, which were the other known *Mollicute* phage hosts to date.

An additional interesting phage of note is the *Acholeplasma* virus L2, type member for the *Plasmaviridae* family. This temperate phage is unique as the progeny virions are released through budding and do not cause lysis of the host, which survives as a lysogen (discussed above) (Gourlay 1971, Gourlay et al. 1973, Krupovic and Consortium 2018). The L2 phage is a dsDNA-enveloped virus with a pseudospherical shape. The viral membrane lipids composition is similar to the host’s and they are organized in a bilayer. It is assumed that infection results from fusion of the membranes; however, this is not fully understood.

Phages capable of infecting *Spiroplasma* were first observed in 1973. As increasing numbers of *Spiroplasma* strains were isolated, it was observed that ‘virus-like particles’ were present in >90% of strains (Liss and Cole 1982). They were characterized into three groups based on morphology: naked rod, short- or long-tailed polyhedron. Of these groups, the naked rod (SpV1) phages are of particular interest as they have a filamentous morphology. They can produce a non-lytic infection similar to *Acholeplasma* group 1 viruses (L2); however, are non-enveloped suggesting extrusion rather than budding.

### Bacteriophage isolation

*Mollicutes* can have complex nutritional requirements, are slow growing, sometimes cannot form a confluent lawn, and can self-toxify (Liao and Chen 1982, Waites and Talkington 2004, Dworkin et al. 2006, Fleming et al. 2021, Rowlands et al. 2021). Indeed, some *Mollicutes* are yet to be cultured and thus have been allocated ‘*Candidatus*’ status. Therefore, isolating and investigating phages with traditional methods in this family is particularly challenging. The earliest *Mollicutes* phages to be isolated were from species including *A. laidlawii, M. bovirhinis*, and *M. hyorhinis* (Gourlay 1970, Gourlay et al. 1983). These bacteria were able to be cultured and the phage visualized with the traditional spot test method. This method involves adding an overnight bacterial broth culture to semi-solid agar, which is poured over an agar plate to achieve a double-layered plate. Once the second layer is dry, a filtrate of interest, which may contain phage is ‘spotted’ onto the plate (Fig. 3a, b, and c). The plate is incubated until a confluent lawn has formed. Phage activity is indicated by zones of clearing or individual plaques. The sample could be from an environmental source such as wastewater or rainwater, or from a human, animal, or plant source. It is also sometimes possible to induce temperate phage from their host by stressing the cells with treatments such as UV irradiation or mitomycin C, or induction may happen spontaneously. The lysate can then be used in spot tests to screen for activity against other bacterial strains.

### Induction of prophage

One of the first *Mollicute* phages (L1) was isolated against *M. laidlawii* (now *A. laidlawii*) by culturing from bovine nasal passages and then cross-spotting filtrates from other *Mycoplasma* cultures (Gourlay 1970). The plates were incubated for 24–48 h at 37°C. All showed confluent lawns apart from one, which had a clear zone of inhibition with a turbid halo. The filtrate was diluted so that single plaques could be observed and were 2–3 mm in diameter (Fig. 3a). The titre was determined to be 2.5 × 10^9^ PFU, with the filtrate demonstrating sensitivity to chloroform, suggesting the virions contained lipids.

*Spiroplasma* group three phages were also isolated using a similar method (Fig. 3b) (Cole et al. 1977). A total of 21 *Spiroplasma* strains were inoculated with filtrates of broth cultures of each other. This resulted in two successful infections from donor strains onto a single *S. citri* recipient host. Activity was observed as large clear plaques after 5 days of incubation at 32°C, 5% CO_2_ and 95% N_2_. Subsequent electron microscopy revealed the type 3 viruses as short-tailed polyhedron virus particles.

Another successful method used to isolate an *A. laidlawii* phage (MV-L2) was to grow a strain on glucose-serum (GS) agar until good colony growth was visible, then wash with 3 mL of phosphate-buffered saline (PBS) for 18 h at room temperature (Gourlay 1971). The wash was then harvested, and the spot test method used to check for phage activity. In this experiment, the filtrate method described above was also used; however, it did not produce any phage activity, unlike the wash method.

A group 1 *Spiroplasma* (SV1) phage was successfully isolated from *S. citri* cultures washed overnight with 10 mL of *Spiroplasma* dilution buffer (Liss and Cole 1981). This phage was also isolated from spotting the supernatant of the *S. citri* host. The virus was purified using CsCl gradient and was highly sensitive to chloroform. Phage activity was observed as turbid plaques. This is interesting to note as these types of phages are filamentous, belonging to the *Plectroviridae* family of ssDNA phages, and do not necessarily kill the host or form true plaques; rather, the bacterial growth is inhibited (Bébéar et al. 1996, Mai-Prochnow et al. 2015). However, for this phage-infected host, cells divided at a rate equal to that of an uninfected culture. Virus release began after 60 min and continued for 6 h.

### Microscopy

There are some *Spiroplasma* that exist as endosymbionts with *Drosophila* flies. This presents a unique challenge when aiming to isolate phages against these bacteria, because *Spiroplasma* cannot be cultured outside of the host. One approach to this issue used transmission electron microscopy (TEM) to visually identify phage-like particles prior to any further sequencing. *Drosophila* containing *S. poulsonii* were homogenized and processed via polyethylene glycol (PEG) precipitation, followed by density step gradient using iodixanol (Ramirez et al. 2022). TEM successfully identified phage-like particles and the subsequent DNA was sequenced. This method identified three phage-like contigs; however, their function remains unknown. TEM can be time consuming and difficult to interpret (e.g. due to artefacts); however, this may be a valid first step when searching for phages in difficult-to-culture bacteria. A collection of further micrographs of *Mollicutes* phages are available in Chapter 14 of The Mycoplasmas V1 (Cole et al. 1979).

### *In silico* bacteriophage identification

An example of true minimalists, some *Mollicute*s have such a reduced genome that they exist on the precipice of maintaining cellular function (Sirand-Pugnet 2007b). The notion of any additional space for phage genomes then becomes an intriguing question and one i.e. important for understanding the fitness trade-offs associated with phages and other mobile genetic elements.

*Mycoplasmas* were used as the model organism for understanding the minimal requirements needed for cellular function (Moger-Reischer et al. 2023). The *M. mycoides* JCVI-syn3B has a synthetically constructed genome reduced from 901 to 493 genes and is the smallest genome of any organism able to be grown in pure laboratory culture (Hutchison et al. 2016, Breuer et al. 2019). This model resulted in understanding of essential genes and subsequently led to the highest reported mutation rate of any cellular organism (3.25  ±  0.16  ×  10^−8^ number of mutations per nucleotide per generation of JCVI-syn3B) (Moger-Reischer et al. 2023).

Alternative codon usage in some *Mollicutes* is important for understanding their genome and provides insight into their evolution. While nearly all organisms use no more or less than the 20 amino acids, the amino acids remain constant; however, the codons are still evolving. The first example of tolerable genetic code changes was observed in mitochondria and were hypothesized to correlate with a small genome (Barrell et al. 1979). Supporting the link to small genomes was the discovery in 1985 of the genetic code (genetic code 4) alteration in Mycoplasmas, that the UGA universal stop codon was read as tryptophan in these organisms (Yamao et al. 1985). These examples challenged the notion of a ‘universal’ genetic codes and highlighted theories on evolution of codon usage, demonstrating those that do not abide by the common code are not merely exceptions to the rule (Ohama et al. 2008).

Phage gene sequences have been identified *in silico* in *Ureaplasma* and *M. hominis* genomes, but no phages have been isolated from these hosts to date. To successfully visualize plaques, one must be able to grow a lawn of the bacteria of interest. This remains a challenge particularly for *Ureaplasma* due to its tendency to self-toxify as bacterial counts increase, and excess ammonia by-products accumulate (Fleming et al. 2021). For this reason, it is also difficult to culture high concentrations of liquid *Ureaplasma* stocks. In lieu of alternative options of phenotypic analyses, whole genome sequencing has provided insight into the presence of phage-like genes and the mobility of genes across species.

Considering the difficulties in isolation, looking for *Mollicutes* phages *in silico* seems a sensible place to start; however, this relies upon good reference databases and bioinformatic tools being available; and knowing how to best use them. This can be a useful approach but also presents a limitation for organisms that are not well studied or for unusual viruses such as *Inoviridae* (Roux et al. 2019). Searching for phages based on homology is reliant on the breadth of the database, which is difficult when there are so few *Mollicute* phage genomes available. One study looked at 82 *M. anserisalpingitidis* genomes by cross-checking several different established bioinformatic platforms, including Prophage Hunter, PhiSpy, and PHASTER, in addition to a novel tool VIBRANT, to search for prophage sequences (Kovács Á et al. 2021). Given the limited database available of *Mollicutes* phage genomes, the approach taken also included a focus on identification of indicators such as differences in G + C content and attachment sites. The findings showed use of the novel software VIBRANT improved results as it not only checks for known phage genes but for proteins, which have functions common in phages and prophages (Kieft et al. 2020). This study highlights that success *in silico* is highly dependent on how we use the tools available (Kovács Á et al. 2021).

There are few *Mollicute* phage sequences available to make homology comparisons; however, the dedicated database MolliGen, aimed to increase the numbers of bacterial genomes and improve annotations (Barré et al. 2004), and further updates on platforms such as this are likely to positively impact the *Mollicute* phage genomics. φMFV1 is a prophage from *M. fermentans* that has been described, but not isolated (Röske et al. 2004). The prophage genome may be related to the MAV-1 phage; however, the hosts are taxonomically distinct. φMFV1 appears to be mobile and encodes a surface protein (mem). Attempts were made to induce φMFV1 to form plaques using the same methods that worked for MAV-1 but were not successful. It has also been reported that the *M. hominis* prophage MHoV-1 is related to φMFV1 and MAV-1 (Calcutt and Foecking 2015).

Repeat regions can also make completing genomes difficult and facilitate genomic rearrangements. *Spiroplasma melliferum* was found to be rich in repeat regions that are suggested to have originated from plectrovirus insertions due to their high similarity to plectroviruses SpV1-C74, SpV1-R8A2B, and SVTS2 (Lo et al. 2013). As mobile elements with the ability to enhance fitness or virulence, prophages have also been associated with antimicrobial resistance genes. In *M. bovirhinis*, a 53 523 bp prophage-like region identified through whole genome sequencing was one of the largest identified in *Mollicutes* and contained aminoglycoside resistance gene cluster *aadE*-*sat4*-*aphA-3* with >99% similarity to *Streptococcus pyogenes* and similarities to *S. suis* phage phi-SsUD.1 (Lysnyansky and Borovok 2021). With the *Mollicute* phages to date described as temperate and many exhibiting chronic lifestyles, it is likely that these phages contribute significantly to this sharing of genes.

### Defence systems in *Mollicutes*

Bacteria have a number of tools to assist in protecting themselves from the constant barrage of external genetic material such as phage attack, including restriction–modification (RM), abortive infection (Abi), and CRISPR systems. All have been identified in several *Mollicute* species (Fisunov et al. 2017). RM systems rely on methylation patterns to detect exogenous nucleic acids entering the bacteria (e.g. phages) and act to cleave unmethylated genetic material to inactivate it. RM systems have been reported in *M. agalactiae* (Dordet-Frisoni et al. 2022), *M. gallisepticum* (Semashko et al. 2022), and *U. parvum* (Wu et al. 2018), among others. The Abi systems, however, act as a self-sacrificing mechanism to limit phage replication by co-ordinating bacterial cell death upon phage infection, suggested as innate bacterial immunity (Lopatina et al. 2020). Two genes have been found in *M. agalactiae* that have products similar to Abi systems found in other bacteria (Citti et al. 2020). Considered an adaptive bacterial immunity, CRISPR/Cas systems provide a defence mechanism for archaea and prokaryotes against invading DNA, particularly viruses, and often act as a time capsule, recording the different phage infections in the bacteria. *Mollicutes* are the bacteria with the smallest genomes in which these systems have been detected to date (Ipoutcha et al. 2019). In a global survey of the 52 representative *Mollicute* genomes, 21 had complete or partial Type II CRISPR/Cas systems (40%). However, they were not detected for the main human pathogens, including *U. parvum* (genomes available, *n* = 14), *U. urealyticum* (*n* = 18)*, M. hominis* (*n* = 23), *M. penetrans* (*n* = 2), and *M. pneumoniae* (*n* = 90) (Ipoutcha et al. 2019). These four human species also happened to include those for which no lytic phages had been isolated to date. A total of 153 genomes for pathogenic human *Mollicutes* were available for the study; however, as additional genomes are added to the database, this should be revisited. CRISPR/Cas and RM type III have also been identified in *M. hyosynoviae* strains. Two out of seven strains contained these defence systems and also had an absence of prophage sequences, whereas prophage sequences were identified in a remaining three strains (Bumgardner et al. 2015). Studying these defence systems will help us to understand the co-evolution of *Mollicutes* and their viruses, as we attempt to uncover whether phages exist in other *Mollicutes* species and what may be hindering phage infection.

## Future directions

Advances in our technology have made it possible to achieve more than ever before. This is an opportunity to explore *Mollicutes* as we have never been able to previously and enhance the field for those understudied species of significant human, animal, and environmental impact. Despite the influx of sequencing data in the current age, given the breadth of *Mollicutes*, further completed genomes will assist in improving research efforts from both bacterial and viral perspectives, as the prophage content can provide valuable primary insight. With the number of difficult and uncultivable *Mollicutes* as the barrier to progress beyond the sequencing data, *in vitro* techniques may be able to replicate the environmental and host niches to sustain growth, e.g. bioreactors (Imachi et al. 2022), microfluidics (Nichols et al. 2010), and organs-on-a-chip (Garcia-Gutierrez and Cotter 2022). The complexity of recreating biological systems can be simplified and reverse engineered to reveal the key requirements for growth. With the foundation of a greater appreciation for the genomic and phenotypic characteristics of *Mollicutes*, phage hunting could be improved.

In contrast, utilizing classical methods, such as fluorescence *in situ* hybridization (FISH), remain a promising solution to visualizing these small organisms that may not be able to be cultured. More specifically, phage FISH combines the sequence knowledge to specifically target and visualize intracellular and free viruses (Allers et al. 2013). Learning from studies on jumbo phages, which have their own set of size-related isolation challenges, metagenomic data from pig microbiome samples were used to detect previously understudied phages and visualize these *in vitro* from the original faecal samples using phage FISH (Ostenfeld et al. 2023). This provides information on where the phages are located and, in this study, demonstrated co-localization of the bacteria and phage signals presenting different stages of the lifecycle (intracellular versus free phage).

The use of fluorescent dyes for detection can also be used via flow cytometry. Flow cytometry enables cells (or particles) to be counted and distinguished based on size and complexity. In the past, resolution of the nanoscale organisms such as *Mollicutes* and viruses using flow cytometry has limited its applications; however, advanced methods appear promising (Brittain et al. 2019, Lawson-Ferreira et al. 2021). Flow cytometry is rapid compared with traditional phage isolation methods and does not rely on visualizing plaques on bacterial lawns. It has been demonstrated that flow cytometry can be used to identify and enumerate *Lactococcus* phage populations (Oliveira et al. 2017), and a similar approach may be useful for *Mollicutes*. Similarly, nanoparticle tracking analysis (NTA) utilizes lasers and fluorescence to count and record particles in a liquid suspension and has successfully been used to enumerate phages (Anderson et al. 2011).

These methods use fluorescent signals as a proxy, while the standard method of virion visualization is via electron microscopy. As mentioned in this review, TEM has been useful to identify viral particles. Enhancing this through the use of cryo-EM could prove extremely useful to understand the structural complexities of *Mollicute* structure, vesicle formation, and phage infection (Stass et al. 2019). The major limitations will be appropriate sample preparation, equipment access, and expertise.

*Mollicutes* are an important class of bacteria that highlight the shortcomings of making assumptions in biology. While we must use our extended knowledge to help to approach and probe new areas, it is vital to remember that sometimes the conventional wisdom does not apply. In phage biology, many of the gold standard techniques of visualization of plaques and phage isolation are those that have been in place for over a century and our information is biased toward those organisms that are suited to these methods. Addressing the ‘difficult’ bacteria will develop a broader understanding and allow for innovative technologies and applications in our field.

## Data Availability

All data and findings referred to in this review are readily available through the referenced original research citations. Data sharing is not applicable to this article as no new data were created or analysed in this review.
